# Pet Companionship Among International Students in the U.S.: Motivations and Challenges

**DOI:** 10.3390/ani15203016

**Published:** 2025-10-17

**Authors:** Jiaqi Tian, Megan K. Mueller, Seana Dowling-Guyer

**Affiliations:** Center for Animals and Public Policy, Cummings School of Veterinary Medicine at Tufts University, North Grafton, MA 01536, USA; megan.mueller@tufts.edu (M.K.M.); seana.dowling_guyer@tufts.edu (S.D.-G.)

**Keywords:** human–animal interaction, international students, companion animals, barriers to pet ownership, motivations for pet ownership

## Abstract

**Simple Summary:**

Over one million international students study in the United States each year, and many feel homesick or stressed while adjusting to a new country. Pet companionship may help alleviate homesickness and support mental well-being, but little is known about the obstacles international students encounter when they have pets or why some choose not to. We surveyed 662 international students to learn why they do or do not have pets in the United States and what challenges they face. Participants reported barriers such as financial and housing restrictions, worries about pet care during travel or vacations, and uncertainty about future plans, which deter long-term pet ownership. Even so, most participants who had pets or planned to acquire one believed the benefits outweighed the challenges. More than half said they would keep their pet for the long term, even if they moved back to their home country or to another country. These findings can help universities, communities, and service providers design better support for students and promote animal welfare.

**Abstract:**

Over one million international students from 207 countries study in the United States to pursue their academic goals. Transitioning to an unfamiliar country presents numerous challenges, and existing support structures often fail to fully support international students. Pet companionship may support students in alleviating homesickness and enhancing mental well-being. However, there is a lack of research exploring the experience of international students in the U.S. living with pets and what unique barriers they face. This quantitative survey recruited 662 international students to explore why they may or may not choose to live with pets while they are in the U.S. and the challenges they face regarding having pets while studying abroad. Participants reported barriers such as financial and housing restrictions, as well as concerns about pet care during travel or vacations and uncertainty about their future plans, which deter them from committing to long-term pet ownership. However, most of the participants who had experience living with pets or planned to have a pet believed that the benefits of having a pet outweighed the challenges. More than 60% of the participants were committed to keeping their pets permanently, even if they needed to move back to their home country or to another foreign country. While results are limited to a non-representative sample of international students, this research provides insights that may inform how to enrich support systems for both international students and animal welfare by highlighting the unique challenges and benefits of human–animal interactions for international students.

## 1. Introduction

### 1.1. Pet Companionship Among International Students in the U.S.: Motivations and Challenges

The United States (U.S.) has been the leading destination for international study for over a century, with 2023–2024 marking another year at the forefront [1,2]. However, this educational journey to an unfamiliar foreign country comes with its share of challenges for international students, including language barriers, culture shock, feelings of isolation and homesickness, as well as experiences of discrimination and prejudice [3,4]. As evidence suggests that pet relationships helped mitigate mental health issues for some people during the pandemic [5], human–animal interactions could also be a potential source of support for international students [6], who often experience similar kinds of isolation [7]. International students could benefit from pet companionship but may face barriers in both acquiring and maintaining pets, as they encounter different obstacles than local students in both academic and daily life [7]. Yet research on these barriers is limited. As numerous factors may inhibit these students from having their own pets or bringing their companions with them while studying in the U.S., there is a need for research focused on understanding the challenges they face in acquiring and maintaining pet companionship during their time abroad. Understanding the desires and obstacles international students face in owning pets could provide institutions across the U.S. with insights into the experiences of these students, leading to the design of more meaningful pet-related policies and regulations.

### 1.2. Challenges for International Students and Benefits of Pet Companionship

In the 2023/24 academic year, over 1.1 million international students from more than 200 countries pursued their academic goals at U.S. higher education institutions [8]. This marked a 7% increase from the previous year, constituting nearly 6% of the total student body in the country and contributing more than $50 billion to the U.S. economy in 2023 [1]. This pursuit, while offering valuable higher education experiences and exposure to different cultures, also presents unique challenges and obstacles for these international students. Among these are similar stressors and developmental challenges that domestic students face upon entering college or university, compounded by the distinct challenges of adapting to a new country and culture [9]. Consequently, these challenges, especially the ongoing need for cultural adjustment, place international students at a heightened risk of experiencing various psychological issues [10]. Homesickness, marked by loneliness, sadness, and adjustment difficulties, is common among international students and often leads to anxiety and depressive symptoms, affecting their academic performance and daily life [4,11]. Existing support structures often fail to fully meet the specific health and well-being needs of international students [12,13]. This inadequacy is highlighted by reports of lower awareness and utilization of mental health services among international students compared to their domestic counterparts [12,14]. A significant factor contributing to this issue is the preference for therapists who speak the students’ native languages, compounded by cultural differences in attitudes toward counseling services. Moreover, the limited availability of therapists with diverse cultural backgrounds frequently leaves these specific needs unaddressed [14].

While pet companionship is not a panacea and cannot substitute for professional mental health services, pets may be an accessible type of social support that can assist in alleviating some symptoms, complementing professional therapeutic interventions [15]. Previous research indicates that social support can mitigate effects of loneliness and homesickness, with a diverse socio-emotional network aiding in psychological well-being and reducing depression and anxiety [16,17]. However, international students often struggle to build such networks due to cultural differences and language barriers, which may lead to increased homesickness [4]. In this situation, pet companionship emerges as a strategy to reduce loneliness since it transcends language barriers. Pets can act as attachment figures, fulfilling a need for closeness and support in their owners. This dynamic can foster an environment where owners are emotionally attached to their pets, providing the emotional support that alleviates stress and evokes positive emotions [18]. Consequently, this nurturing relationship enhances the mental well-being of pet owners by addressing the fundamental human need for belonging and love, which is particularly significant for international students [16,18,19]. Although there are distinctions between human–human and human–animal interactions, pets can partially fulfill a person’s social needs, offering a form of social support linked to positive mental health outcomes, especially when isolated from human contact [20,21]. Research has found that individuals highly attached to their pets often perceive little difference between human–human and human–animal interactions and that pet companionship can serve as a complement to other sources of social support [19,22]. Pet interactions have been shown to alleviate student homesickness and enhance daily and campus life satisfaction, which can be one useful strategy to help students [23]. Previous research has shown that since the COVID-19 lockdown, pet owners experienced smaller decreases in mental health and smaller increases in loneliness compared to non-pet owners [5,24]. This supports the notion that pet ownership can serve as an effective coping strategy for managing isolation and loneliness in situations where human interaction is limited. This is particularly beneficial for international students, helping them reduce loneliness and maintain their mental well-being, especially for those struggling with human relationships.

### 1.3. Obstacles in Acquiring and Maintaining Pet Companionship

International students seeking pet companionship may face numerous barriers that inhibit both the acquisition and maintenance of pets while studying abroad. Current literature fails to adequately address the key factors that deter international students from pet ownership and the unique challenges they face while attempting to own pets during their studies abroad. In addition to common issues such as housing restrictions and financial burdens, international students face added complexities such as future uncertainties and less social support [11] not to mention that international students must also consider the feasibility of transporting pets back to their home countries after their studies. This requirement may force difficult decisions due to policies or obstacles that may hinder their ability to maintain this commitment. Transporting pets involves navigating policy restrictions, including breed import limitations and health and vaccine requirements, which can be formidable when coupled with substantial transportation costs and the financial impact of fluctuating currency exchange rates [25].

Previous research suggests that international students may benefit significantly from pet companionship during their U.S. studies [11,23,24], highlighting the urgent need to explore the obstacles they face acquiring and maintaining a pet. However, there is little research exploring how the cultural, legal, and economic contexts of these students influence their challenges and needs concerning pet ownership. This lack of knowledge limits support for international students, hindering their ability to benefit from human–animal interactions that provide emotional support, aid in cultural integration, and foster personal development. Further research is necessary to develop comprehensive support systems that address their specific needs for companionship and comfort. Early identification of these barriers is critical to preparing students for the challenges of pet ownership and reducing pet relinquishment rates.

### 1.4. Current Study

To address the gap in the literature concerning the multifaceted challenges international students face in acquiring and maintaining pet ownership in the U.S., this study explored the challenges and obstacles students encountered while trying to acquire and maintain pets during their studies. This descriptive online survey aimed to uncover the underlying motivations for and barriers to pet ownership for U.S. international students, providing a deeper understanding of this aspect of their experience abroad.

## 2. Method

All procedures for this study were approved by the Institutional Review Board at Tufts University to ensure human safety.

### 2.1. Participants

A total of 763 eligible participants were recruited through social network posts and direct assistance from 23 universities across the U.S. Eligibility criteria for participating in the study included (1) being 18 years old or older, (2) currently residing in the U.S., (3) being enrolled as a full-time student with an F-1 visa (excluding students in Optional Practical Training who are not full-time students), and (4) being non-resident aliens or resident aliens, meaning they must be citizens of countries other than the U.S. (excluding U.S. permanent residents or individuals with dual citizenship that includes U.S. citizenship). Following data collection, the inclusion criteria for data analysis required participants to pass authenticity and attention checks that were included in the questionnaire; 101 participants were excluded for failing these checks.

The final analytic sample consisted of 662 international students. Participant age ranged from 18 to 55 years, *M* = 25.6 (*SD* = 5.2). Participants reported the year they first arrived in the U.S. to study; the time since their initial arrival ranged from 1 to 16 years, *M* = 3.9 (*SD* = 3.1). Participants came from the following 18 different regions: East Asia (*n* = 187, 28.2%), Southeast Asia (*n* = 83, 12.5%), South Asia (*n* = 181, 27.3%), Central Asia (*n* = 16, 2.4%), Middle East (*n* = 44, 6.6%), North Africa (*n* = 5, 0.8%), West Africa (*n* = 7, 1.1%), Sub-Saharan Africa (*n* = 18, 2.7%), Western Europe (*n* = 19, 2.9%), Eastern Europe (*n* = 16, 2.4%), North Europe (*n* = 4, 0.6%), South Europe (*n* = 10, 1.5%), Canada (*n* = 17, 2.6%), Mexico (*n* = 6, 0.9%), the Caribbean Islands (*n* = 5, 0.8%), Central America (*n* = 4, 0.7%), South America (*n* = 34, 5.1%) and Oceania (*n* = 7, 1.1%).

### 2.2. Procedure

Identifying information about the participants was not collected. The study used convenience sampling, targeted sampling, and snowball sampling. Recruitment information was posted to international student groups on social media platforms such as Facebook, Instagram, Reddit, LinkedIn, and RedNote. To recruit targeted participants, we also contacted 206 universities’ International Student and Scholar Services (ISSS) or other international student institutions to distribute the survey invitation among both their undergraduate and graduate international students across the U.S. Of these, 63 universities responded to the request, with 23 agreeing to assist; others may have distributed the invitation without responding directly. A link in the recruiting posts and emails directed potential participants to the anonymous, online survey hosted on Qualtrics, an online survey platform. Participants were offered the chance to enter a drawing for one of five $50 gift cards at the end of the questionnaire. Completing the questionnaire took 5 to 15 min. The questionnaire was available from 11 July to 30 November 2024.

### 2.3. Measures

#### 2.3.1. Demographics and Housing

Participants were asked to self-report standard demographic questions such as age and gender identity, as well as degree they are currently pursuing, first year arrived in the U.S. for studying, current state location, and region of origin. Participants reported their living situations including university-organized rental housing, non-university-organized rental housing, privately owned off-campus housing, and host family accommodations, as well as whether participants currently live with others.

#### 2.3.2. Pet Ownership

Participants were asked to report their pet ownership during their study period in the U.S., where they acquired their pets, whether the pet was an emotional support animal (ESA) or not, type of pets (dog, cat, small mammal, reptiles or amphibians, fish, bird, horse or other), number of each type of pet, and why they were interested in owning pets during their study period in the U.S.

#### 2.3.3. Obstacles to Pet Ownership

Obstacles related to acquiring and maintaining pets were divided into two categories: obstacles to acquiring pets and obstacles to maintaining pets. Only participants who currently owned pets, owned pets in the past, or planned to own pets in the future during their study period in the U.S. were eligible to respond to these sections. Items were completed using 5-point Likert items (1 = strongly disagree, 5 = strongly agree). Participants who selected “6 = prefer not to answer” were excluded from the analysis of that item. Lower values indicate that participants were less likely to perceive the items as obstacles. The section on acquiring pets contained seven questions (e.g., housing restrictions, sellers or shelters less willing to sell/adopt to me because I identified as an international student) while the section on maintaining pets included 11 questions (e.g., financial burden, my uncertainty about future plans prevents me from committing to long-term pet ownership). An open-ended text box was provided for participants to share any additional thoughts related to the obstacles they had encountered in owning pets in the U.S.

International students who did not plan to own pets during their current or future study periods in the U.S. were presented with a multiple-choice question. This question listed eight different reasons for not planning to own pets (e.g., dislike for pets by me or others living with me, future uncertainty affects long-term commitment) participants could select from and provided an option for participants to list any other reasons they preferred to share.

#### 2.3.4. Future Plans for Pet Ownership

Maintaining pets during the study period in the U.S. represents only one aspect of the challenges to continued pet ownership for international students. To further understand their specific obstacles, questions were included about international students’ plans for their pets after completing their studies. This section was applicable only to participants who currently owned pets or planned to own pets in the future. It featured questions about their plans for their pets when they return to their home country or move to another country. Additionally, participants who were uncertain about their future plans regarding their pets, and who might intend to leave some or all of their pets in the U.S., were asked to identify up to three main reasons from a list of nine for potentially parting with their pets. They were also asked to choose their plans for any pets they decide not to bring with them when they return to their home country. Additionally, participants were given the option to list any additional reasons and future plans for pets they wished to share.

#### 2.3.5. Attitudes Towards and Perceived Benefits of Pet Ownership

Two rating scale questions assessed the extent to which participants agreed with the statements “International students should avoid having any pets (all species) during their study period in the U.S.” and “The benefits of having a pet outweigh the challenges.” The initial question was open to all participants. However, the second question was specifically designed for those who currently owned pets, previously owned pets, or planned to own pets in the future. Items were completed as 5-point Likert items (1 = strongly disagree, 5 = strongly agree). An open-ended text box was provided for participants to share any additional thoughts related to pet ownership in the U.S., regardless of the area or aspect they wished to mention.

### 2.4. Data Analysis

All analyses were performed in SPSS version 29.0. Pet ownership was categorized into four mutually exclusive groups: (1) currently owning pets, irrespective of past or future plans; (2) not currently owning pets but having owned them in the past, without consideration of future plans; (3) never owned pets but plan to do so in the future; and (4) neither owned pets in the past nor plan to own pets in the future. Descriptive statistics (mean, median, standard deviation) were calculated for numeric questions such as age and years in the U.S. Frequencies were calculated for all categorical questions. Descriptives and frequencies were stratified by pet ownership group. Because the 5-point Likert items, which were used with both the seven questions on obstacles to acquiring pets and the 11 questions on maintaining pets, were not normally distributed, the Kruskal–Wallis test was calculated to compare responses across different pet ownership groups. The two items regarding attitudes and perceived benefits of pet ownership were not normally distributed; therefore, the Kruskal–Wallis test was also used to test differences in agreement ratings between different pet ownership groups. Mean ranks were used because the distributions for each pet ownership group were not similar, as assessed through visual inspection of the histograms. For significant Kruskal–Wallis tests, post hoc pairwise comparisons were conducted to identify group differences. The question regarding participants’ future plan for pets was assessed using a Pearson’s Chi-square test to explore its association with participants’ pet ownership status, with post hoc z scores used to determine which groups differed significantly from each other. All the expected counts were larger than 5. For all comparisons, *p* values < 0.05 were considered statistically significant. We calculated eta squared (*η*^2^) and Cramer’s *V* as indices of effect size. These can be interpreted as 0.01 = small, 0.06 = medium, and 0.14 = large for *η*^2^ and 0.10 = small, 0.30 = medium, and 0.50 = large for Cramer’s *V* [26].

Responses to the open-ended text box items in the questionnaire were reviewed and descriptively analyzed to supplement the quantitative findings related to participants’ experiences, motivations, and obstacles regarding pet ownership in the U.S.

## 3. Results

### 3.1. Demographics and Pet Ownership

Of the 662 participants, 149 students (22.5%) reported currently owning pets, 31 (4.7%) did not currently own pets but owned them in the past, 233 (35.2%) had never owned pets but planned to do so in the future, and 249 (37.6%) neither had owned pets in the past nor planned to own pets in the future. Participants resided in 36 different states, most frequently from New Jersey (189, 28.5%) and Texas (130, 19.6%). Among the participants who currently owned pets, 36.9% (*n* = 55) reported that their currently owned pets were emotional support animals (ESAs). Additionally, 19.4% (*n* = 6) of participants who had previously had a pet indicated that their pets were ESAs, and 19.3% (*n* = 45) of participants who planned to own pets in the future stated that their pets will serve as ESAs. Demographic and contextual indicators stratified by pet ownership status are presented in Table 1.

Of the 180 international students who either currently own or have owned pets while studying in the U.S., 108 (60%) had cats, 66 (37.6%) had dogs, 17 (9.4%) had small mammals (e.g., rabbits, guinea pigs, hamsters), 5 (2.8%) had reptiles or amphibians, 12 (6.7%) had fish, 2 (1.1%) had birds, and 6 (3.3%) had other species of pets; multiple responses were allowed since participants could own pets of different species. Of the 233 participants who currently do not own pets but plan to do so while studying in the U.S., 126 (54%) international students intend to own cats, 155 (66.5%) plan to own dogs, 19 (8.2%) plan to own small mammals (e.g., rabbits, guinea pigs, hamsters), 9 (3.9%) plan to own reptiles or amphibians, 30 (12.9%) plan to own fish, 17 (7.3%) plan to own birds, 9 (3.9%) plan to own horses, and 1 (0.4%) plans to own other species of pets (multiple-response question).

Among those international students who currently owned pets or owned pets previously (*n* = 180), 41.1% (*n* = 74) adopted their pets from a U.S. shelter or rescue organization, 34.4% (*n* = 62) purchased their pets from a pet breeder, local pet store, or personal seller in the U.S., 20.0% (*n* = 36) brought pets from their home country, 9.4% (*n* = 17) received their pets from someone in the U.S. who is not an international student and needed to find a new home for their pets, 8.9% (*n* = 16) obtained their pets from another international student who needed to rehome their pet in the U.S., 6.1% (*n* = 11) received their pets as gifts, and 5.6% (*n* = 10) found their pets as strays and adopted them in the U.S. (multiple responses allowed).

For participants who currently did not have pets but planned to acquire them (*n* = 233), 85.0% (*n* = 198) indicated they might adopt pets from a U.S. shelter or rescue, 37.3% (*n* = 87) might purchase pets from a pet breeder, local pet store, or personal seller in the U.S., 35.6% (*n* = 83) prefer to obtain pets directly from non-international students needing to rehome their pets in the U.S., 35.2% (*n* = 82) would like to adopt pets directly from other international students who need to rehome their pets, 30.9% (*n* = 72) want to find and adopt stray pets in the U.S., and 16.7% (*n* = 39) plan to bring pets from their home country to the U.S. (multiple responses allowed).

### 3.2. Motivations for and Obstacles to Pet Ownership

Among the 413 international students who currently owned pets, previously owned pets, or planned to own pets in the future, 81.6% (*n* = 337) indicated in a multiple response question that their motivation for owning pets was to enjoy the love of animals and their company. Additionally, 67.3% (*n* = 278) reported seeking companionship or emotional support as their motivation; 53.5% (*n* = 221) said they owned pets to alleviate feelings of loneliness or homesickness; 43.3% (*n* = 179) are motivated by the assistance pets provide with mental health issues; 37.8% (*n* = 156) by the opportunity to gain a sense of responsibility and routine; 28.3% (*n* = 117) to improve their physical activity and health; 18.6% (*n* = 77) to connect with others and build a community; 17.2% (*n* = 71) to avoid separation from their hometown pets by bringing them to the U.S.; and 7.7% (*n* = 32) were motivated by the desire to experience a culture different from their home country.

Participants who did not currently own pets but owned them in the past selected various reasons for why they no longer have their previous pets in a multiple response question. The most common reasons included the death of their pets (8, 25.8%), difficulty balancing pet care with other responsibilities (8, 25.8%), and financial constraints (8, 25.8%). Other reasons were that they either took them back to their home country and did not bring them back (5, 16.1%), their ex-partner took the pets after a breakup (5, 16.1%), they could not keep their previous pets because their current living arrangements prohibit pets (4, 12.9%), health concerns for themselves or others (2, 6.5%), the pet’s behavioral issues (1, 3.2%), or a loss of interest in keeping pets (1, 3.2%). Lastly, 12.9% (*n* = 4) noted in the text box that they were temporarily caring for these pets while the original owners were out of the U.S., and all these pets have since returned to their original owners.

In a multiple-response question answered by international students who have neither owned pets in the past nor planned to in the future (*n* = 249), 73.9% (*n* = 184) cited financial burden as the reason for not planning to own pets during their study period in the U.S. Additionally, 63.1% (*n* = 157) mentioned difficulty in balancing pet care with other responsibilities, such as studies; 62.2% (*n* = 155) expressed concerns about pet care during international or domestic travel; 55.4% (*n* = 138) were uncertain about their future and felt unable to commit to a long-term relationship with pets; and 49.4% (*n* = 123) faced housing restrictions that limited pet ownership. Furthermore, 15.7% (*n* = 39) pointed to health concerns for themselves or others residing with them as a deterrent, while 14.1% (*n* = 35) noted lack of support from parents or friends. Lastly, 11.6% (*n* = 29) stated that they did not like pets or lived with someone who does not. For the other 413 international students who currently owned pets, owned pets before, and planned to own pets in the future, descriptive statistics for the ratings of obstacles to acquiring pets in the U.S. are presented in Table 2, and descriptive statistics for the ratings of obstacles to maintaining pets in the U.S. are presented in Table 3.

In addition to the quantitative results, some participants also mentioned obstacles to pet ownership in the U.S. that were not included in the multiple-choice options, such as language barriers and cultural differences. One participant specifically noted that they were not familiar with medical terminology even in their native language, making it even more difficult to describe symptoms clearly or understand veterinarians easily in English. Another participant pointed out that compared to their home country, the U.S. has stricter policies concerning issues such as neighborhood noise regulations and pet welfare, such as vaccination requirements and laws prohibiting dogs from being left in cars. These stricter policies were described as a source of stress for them as pet owners. However, it is important to note that not all cultural differences were perceived as obstacles. One participant mentioned that pet-related resources in the U.S., such as pet food and veterinary care, were more widely available and accessible than in their home country. This availability encouraged them to consider owning a pet while studying in the U.S.

### 3.3. Future Plans for Pets

Among all participants who currently owned pets and those planning to own pets in the future, 380 responded to the section about their future plans for their pets upon returning to their home country or moving to another country outside the U.S. (see Table 4).

Among the 148 participants who intended to leave some or all of their pets in the U.S. or were uncertain about their future plans for their pets, the top three reasons cited for not definitively planning to bring their pets with them were: 59.5% (*n* = 88) mentioned that considering the stress of long-distance transportation, they believe rehoming might be a kinder option for their pets; 56.8% (*n* = 84) indicated that the paperwork for transportation and importation certifications is too complicated, which deters them from bringing their pets; and 49.3% (*n* = 73) noted the significant costs associated with transporting their pets back home. Additionally, 48.0% (*n* = 71) reported that current transportation rules restrict them from bringing their pets on board; 26.4% (*n* = 39) chose reasons related to their home country’s restrictions on importing animals of their pets’ species; 25.0% (*n* = 37) stated that people they will live with, or their landlord in their home or another country, are not comfortable with pets; and 10.1% (*n* = 15) mentioned that their existing pets at home may not get along with the new ones. Furthermore, regarding their future plans for their pets, 64.9% (*n* = 96) reported that they would personally find a new home for their pets, 11.5% (*n* = 17) stated that they would bring their pets to a local shelter or rescue, and 20.2% (*n* = 30) indicated that they did not have a plan yet.

### 3.4. Attitudes Towards and Perceived Benefits of Pet Ownership

Overall, the results indicate a fairly neutral stance among participants when asked if international students should avoid having pets. There was a significant difference in the beliefs about whether international students should avoid having pets between groups of students (see Table 5). Specifically, current pet owners were less likely to believe that international students should avoid having pets. Compared to participants who planned to own pets, those who did not plan to own pets were less likely to believe that international students should have pets. Also, participants had a tendency to agree that the benefits of having a pet outweighed the challenges. Again, there was a significant difference among the groups in their responses to this statement (see Table 5). Participants who currently owned pets in the U.S. were more likely to agree that the benefits of having a pet outweighed the challenges, compared to the other two groups.

A total of 75 comments were received through the final open-ended text box, in which participants were invited to share their thoughts on the two agreement statements and the study as a whole. Many comments focused on advocating for the benefits of pet ownership among international students. Several participants emphasized that, regardless of cultural background or student status, a sense of responsibility is the most important aspect of being a pet owner. One participant noted that while pet companionship is undoubtedly beneficial, a mutually beneficial relationship requires effort and commitment from the owner. Additionally, some participants expressed concern that certain pet owners, both current or prospective, may underestimate the responsibilities involved in pet ownership, often due to a lack of prior knowledge or access to relevant resources. A few respondents also shared that, because of the many barriers they faced to owning a pet themselves, they chose to volunteer at animal shelters in order to experience the emotional benefits of being around animals. These participants reported that even temporary interaction with animals during volunteer work had a positive impact on their well-being.

## 4. Discussion

The results of this study revealed both the motivations for and the obstacles to pet ownership among international students during their studies in the U.S. The top two motivations for international students to own pets were to enjoy the affection and companionship of animals and to seek emotional support. Concerns about pet care during travel were the primary obstacles to pet ownership among international students in the U.S. Notably, most participants intended to bring their pets with them if they were to return to their home country or move to another country outside the U.S. This finding highlights their sense of responsibility as pet owners and underscores the strong bond they feel with their pets. Furthermore, the high level of agreement with the statement that the benefits of having a pet outweigh the challenges, with a relatively high agreement amongst current pet owners, reinforces the value of pet companionship and human–animal interaction. These findings suggest a need for further research into how pets benefit international students, in which areas these benefits are most evident, and how effective such companionship is.

Alleviating loneliness was one of the most common reasons participants in this study reported as a motivation for owing pets, which was similar to those of general college students [21]. Our study found international students’ motivation for owning pets was also strongly influenced by the desire for companionship and the feeling of being loved by pets, which is a similar to prior research [21,27]. Our results support the idea that pet companionship can serve as a coping strategy for international students who are separated from family and friends, as well as from a familiar environment [23]. This support system provided by pets not only helps in alleviating feelings of isolation but also enhances overall well-being by fulfilling emotional and social needs during their time abroad [22]. A deeper connection with pets can enable social support for pet owners similar to that provided by human contacts [19,28]. However, it is important to note that incongruent expectations about the role of pets can negatively impact the animal’s welfare, and the pet’s own health concerns may cause student owners to be more stressed [15]. Therefore, it is important for international students to understand the realities of pet ownership and manage their expectations before acquiring a pet, to avoid impractical expectations.

Financial burden was the most frequently noted reason international students did not plan to own pets during their study period in the U.S. It also posed a significant obstacle to acquiring and maintaining pets among these students, consistent with their higher reports of financial problems [12]. The costs associated with pet ownership, including veterinary care, general expenses, and additional rental fees for pet owners, present significant initial obstacles. Although financial concerns are not the primary reason the U.S. general public cites for not having pets [29], these costs, especially veterinary care, are noteworthy barriers [30]. For international students, studying in the United States requires spending in U.S. dollars, due to the exchange rates, which can significantly increase their living expenses compared to studying and caring for pets in their home countries. In addition to typically paying higher tuition fees than domestic students, international students also face employment restrictions that limit their ability to earn income, resulting in a greater financial burden [31,32]. In our study, most international students were aware that pet ownership would incur substantial costs. However, even if students use online pet cost calculators [33], these calculators often include a limited range of expenses and may not reflect the specific financial challenges faced by international students, such as international transportation fees and temporary pet care arrangements during travel. As a result, these tools may lead to an underestimation of the true cost of pet ownership. Therefore, improvements in pet cost calculators tailored to the needs of international students would be beneficial, helping to provide more realistic cost estimates and enabling students to make informed decisions about whether pet ownership is financially feasible.

Housing restrictions also represented significant obstacles for international students owning pets. Research shows that housing restrictions account for 14% of pet relinquishments to shelters in the U.S. [34]. Many colleges, especially those housing freshmen and international students, mandate residency in student dormitories that typically prohibit pets such as cats and dogs. In the general housing market, there is no legal protection for pet ownership (except service animals and emotional support animals through the Fair Housing Act), meaning that landlords have the legal right to refuse tenants with pets [35]. Additionally, rental regulations vary by state, and individual housing units may impose their own policies regarding pet fees, deposits, and monthly pet rent. All these extra costs and uncertainties combined together may create significant barriers for international students having pets. Further regulation of pet-related charges in the U.S. housing market may be necessary to better support access to pet ownership with both the associated financial burdens and housing restrictions. As the Fair Housing Act (FHA) protects tenants’ rights to keep ESAs, students may be allowed to own pets once they meet the necessary criteria set by universities and off-campus housing providers [35]. Therefore, individuals living with mental health disorders who seek pet companionship may consider ESAs. So, it is worth broader dissemination and education on this topic.

Having trouble balancing pet care with other responsibilities, concerns about pet care during travel, and uncertainty about future plans were also highly rated obstacles that prevent international students from committing to long-term pet ownership. Moving within the U.S. is already one major reason why the general public is no longer able to keep their pets [36]. For international students, this issue is compounded by the need to consider not only relocation within the U.S. but also international moves after completing their studies, whether returning to their home country or moving to another country. There are many restrictions on the international transportation of pets. For example, most airlines allow only dogs and cats to be transported by air, with some extending this policy to include household birds. Other species are generally prohibited, and certification requirements for importing pets vary across countries [37]. These restrictions together create a substantial logistical and financial burden for those transporting their pets from the U.S. to their home country or another destination. Providing information on traveling with pets to international students in advance may help students anticipate obstacles and better understand the long-term responsibilities of pet ownership, ultimately reducing the risk of future relinquishment and promoting animal welfare. Especially, it is important to note that one third of the participants expressed uncertainty about their future plans regarding their pets, a considerable proportion that raises concern for their pets’ welfare. This finding highlights the need to inform international students about the potential uncertainties of pet ownership before they decide to acquire a pet.

Pet ownership status was a predictor of attitudes about whether international students should have pets and the perceived benefits of pet ownership. Current pet owners were more likely to agree that international students should have pets if they wished regardless of their status and that the benefits of pet ownership outweighed the associated challenges. This aligns with previous research findings, which suggest that providing support for a close other can itself be a source of need satisfaction, fostering well-being, decreasing distress, and enhancing closeness [38]. Consequently, pet owners may benefit from satisfying their pets’ needs, and a deeper connection with their pets may lead to better mental well-being [19,22]. For international students, who often experience limited access to social support [17], the bond with their pets may be stronger than that of the general population. Future research should further explore this reciprocal relationship to better understand how pet owners’ psychological well-being is influenced by their interactions with their pets and whether reduced human–human interaction may lead to greater emotional investment in human–animal interaction. In addition to the documented benefits, previous studies have found that high levels of emotional disclosure to pets are also associated with increased risks of loneliness and depression among youth [39], and deeper connection with pets made them more likely to rely on their pets for companionship and support [22]. This relationship should also be examined among international students to determine whether a deeper emotional bond with pets may lead to similar negative outcomes. International students represent a particularly relevant and valuable population for future research on these questions. Investigating this dynamic could provide valuable insights into the complex interplay between human and animal well-being, potentially guiding interventions that enhance the lives of both.

The current findings indicate that international students who are interested in animals often look forward to experiencing pet companionship during their studies in the U.S. However, as this study has identified numerous obstacles to both acquiring and maintaining pets, alternative opportunities for animal interaction should be considered and made available to students who wish to engage with animals. For example, programs providing visitation with trained therapy animals on university campuses have been shown to improve students’ mental well-being [23,40,41]. These activities should be more widely implemented and offered with greater frequency to ensure that students who are interested have increased access to opportunities for pet companionship.

Furthermore, future research is needed to explore how animal shelters can collaborate with universities to offer both volunteer opportunities and pet fostering opportunities for international students. As noted by some participants in this study, volunteering at animal shelters fulfilled their need to be around animals, especially for those who were not in a position to own a pet. Although fostering pets can provide international students with companionship without the burden of long-term commitment, this approach is often hindered by housing restrictions and difficulties in balancing pet care with academic and personal responsibilities and should be considered carefully.

### Limitations and Future Directions

A key limitation of this research relates to the representativeness of the participants. Overall, the sample recruited too few male international students compared to the general population of male international students [42]. Also, the sample included a higher number of international students who are currently pursuing doctoral or professional degrees in the U.S., a similar rate of students pursuing master’s degrees, and fewer students pursuing bachelor’s or associate degrees compared to the actual population [43]. Based on the actual population’s places of origin, we have recruited disproportionately many participants from Southeast Asia and comparatively fewer from East, South, and Central Asia, while reporting similar rates for other regions of origin [44]. Compared to U.S. households, international students in the U.S. are more likely to own cats and less likely to own dogs, with little variation in the ownership of other species [45]. Collectively, these issues contribute to a sample that is not adequately representative of the broader population of the whole international community. International students who feel a stronger connection with their pets may be more likely to participate in this research, which could influence the results. This research was limited to international students who are currently full-time students under an F-1 visa and did not address other types of visa statuses. Future studies employing more representative sampling strategies will be necessary to validate and extend these findings. Potential directions include adopting sampling methods beyond convenience sampling and embedding pet-related questions within broader surveys that are less likely to disproportionately attract individuals with a preexisting interest in pets.

Furthermore, different species of pets may lead to varying challenges for international students in acquiring and maintaining pets. Other variables, such as current state of residency and length of time spent in the United States, as well as differences both within and between pet ownership status groups, may also influence international students’ propensity to own pets and the obstacles they encounter. However, as this study was descriptive, it did not further analyze these covariates. Future research could explore these variables to develop a more complex analysis and gain a deeper understanding of the barriers that inhibit international students from owning pets and maintaining long-term relationships with them.

There were also some limitations related to the measurement tools. Since the questionnaire was developed solely in English, language barriers should be acknowledged as a potential limitation, as they may have affected participants’ comprehension and response accuracy. Another limitation is that we did not provide a formal definition of “ESA” in the questionnaire. As a result, participants may have used the term informally to describe pets that support them emotionally, which may not align with the U.S. legal definition but may better reflect how people commonly use the term. In addition to these concerns, because most of the survey questions were presented in a multiple-choice format, participants were restricted to selecting from a predetermined set of responses. This limited the flexibility of their answers and may have constrained the expression of their true perspectives. Notably, several participants used the open-ended text box to indicate that none of the provided options fully captured their experiences or opinions.

Regarding language-related obstacles, existing research had primarily focused on immigrant populations in the U.S. [46]. These studies have led to efforts such as encouraging greater diversity among veterinary students and staff to gradually reduce language barriers. Although these initiatives were developed with immigrants in mind, they may also be applicable to international students, given the similarities in linguistic and cultural challenges. However, there is still limited research examining how cultural differences may increase the obstacles to pet ownership in the U.S. Participants expressed a lack of familiarity with U.S. pet ownership culture and voiced concerns about inadvertently violating these unfamiliar rules, potentially leading to illegal acts or even lawsuits. Future research should explore how cultural differences exacerbate the challenges of pet ownership and care across different ethnic groups. 

## 5. Conclusions

Overall, the results of this study indicated that international students were primarily motivated to own pets for companionship, to experience the feeling of being loved by animals, and to alleviate loneliness and homesickness. Similarly to the general population, financial burdens and housing restrictions were major obstacles to pet ownership. In addition, international students faced unique challenges such as uncertainty about their future plans and concerns about finding pet care during international travel. Nevertheless, the findings showed that most international student pet owners were committed to maintaining long-term relationships with their pets regardless of future circumstances. Furthermore, pet ownership status emerged as a significant predictor of international students’ attitudes toward pet ownership and their perceived benefits of having pets.

It is essential for international students to be informed about the potential obstacles they may face before deciding to own a pet during their study period in the U.S. Therefore, providing pre-ownership education that highlights the challenges of pet ownership and difficulties in acquiring and maintaining pets is highly recommended. In addition, alternative strategies such as volunteering at animal shelters or participating in university-based animal-assisted therapy and animal visitation programs may offer valuable opportunities for pet companionship without the long-term commitment of ownership. All of these resources may help international students better understand the responsibilities associated with pet ownership, prevent unmanageable challenges, and reduce the risk of pet relinquishment. Ultimately, the goal is to promote student well-being while also safeguarding animal welfare.

## Figures and Tables

**Table 1:** Demographic Characteristics of the Participants by Pet Ownership Status.

	Currently Own Pets	No Pets, Owned Pets Before	No Pets, Plan to Own Pets	No Plan to Own Pets	Total
	*n* = 149	*n* = 31	*n* = 233	*n* = 249	*n* = 662
Demographic/Contextual Indicators	*M* (*SD*)	*M* (*SD*)	*M* (*SD*)	*M* (*SD*)	*M* (*SD*)
Age	27.43 (5.83)	26.03 (4.45)	24.86 (4.73)	25.05 (5.17)	25.6 (5.2)
Years Since First Arrival in the U.S. for Studying	5.60 (3.52)	6.24 (3.33)	3.02 (2.3)	3.35 (2.75)	3.9 (3.1)
Gender	*n* (*%*)	*n* (*%*)	*n* (*%*)	*n* (*%*)	*n* (*%*)
Cisgender Female	96 (64.4%)	15 (48.4%)	144 (61.8%)	126 (50.6%)	381 (57.6%)
Cisgender Male	36 (24.4%)	13 (41.9%)	58 (24.9%)	78 (31.3%)	185 (27.9%)
Transgender Female	1 (0.7%)	0 (0%)	0 (0%)	0 (%)	1 (0.2%)
Transgender Male	0 (0%)	0 (0%)	1 (0.4%)	6 (2.4%)	7 (1.1%)
Non-binary/gender-fluid or other	6 (4.0%)	2 (6.5%)	5 (2.1%)	14 (5.6%)	27 (4.1%)
Prefer not to say	7 (4.7%)	1 (3.2%)	21 (9.0%)	23 (9.2%)	52 (7.9%)
Current Degree	*n* (*%*)	*n* (*%*)	*n* (*%*)	*n* (*%*)	*n* (*%*)
Associate’s Degree	0 (0%)	0 (0%)	2 (0.9%)	6 (2.4%)	8 (1.2%)
Bachelor’s Degree	22 (14.8%)	10 (32.3%)	59 (25.3%)	72 (28.9%)	163 (24.6%)
Master’s Degree	28 (18.8%)	11 (35.5%)	76 (32.6%)	94 (37.8%)	209 (31.6%)
Doctoral Degree or Professional Degree	99 (66.4%)	10 (32.3%)	96 (41.2%)	77 (30.9%)	282 (42.6%)
Type of Housing Currently Living	*n* (*%*)	*n* (*%*)	*n* (*%*)	*n* (*%*)	*n* (*%*)
University-Organized Rental Housing	13 (8.7%)	6 (19.4%)	58 (24.9%)	70 (28.1%)	147 (22.2%)
Non-University-Organized Rental Housing	124 (83.2%)	23 (74.2%)	158 (67.9%)	153 (61.4%)	458 (69.2%)
Privately Owned Off-Campus Housing	10 (6.7%)	2 (6.5%)	12 (5.2%)	20 (8.0%)	44 (6.6%)
Host Family Accommodations	2 (1.3%)	0 (0%)	5 (2.1%)	6 (2.4%)	13 (2.0%)
Currently Live with Other People *n* (*%* yes)	109 (73.2%)	24 (77.4%)	192 (82.4%)	226 (90.8%)	551 (83.2%)
Pet Ownership in Home Country	*n* (*%*)	*n* (*%*)	*n* (*%*)	*n* (*%*)	*n* (*%*)
No Pet	28 (18.8%)	4 (12.9%)	46 (19.7%)	67 (26.9%)	145 (21.9%)
Cat	56 (37.6%)	14 (45.2%)	78 (33.5%)	74 (29.7%)	222 (33.5%)
Dog	75 (50.3%)	18 (58.1%)	122 (52.4%)	121 (48.6%)	336 (50.8%)
Small Mammal	37 (24.8%)	6 (19.4%)	33 (14.2%)	49 (19.7%)	125 (18.9%)
Reptiles or Amphibians	12 (0.8%)	4 (12.9%)	19 (0.8%)	11 (4.4%)	46 (6.9%)
Fish	44 (29.5%)	11 (35.5%)	64 (27.5%)	58 (23.3%)	177 (26.7%)
Birds	23 (15.4%)	10 (32.3%)	38 (16.3%)	40 (16.1%)	111 (16.8%)
Horse	5 (3.4%)	2 (6.5%)	7 (3.0%)	1 (0.4%)	15 (2.3%)
Farm Animals as Pets	0 (0%)	0 (0%)	3 (1.3%)	2 (0.8%)	5 (0.8%)

Note: *n*: frequencies; *%*: percentage; *M*: mean; *SD*: standard deviation.

**Table 2:** Descriptive Statistics for Ratings of Obstacles to Acquiring Pets in the U.S. by Pet Ownership Status.

	Pet Ownership Status									Total *n* = 413			Kruskal–Wallis Test Results	Effect Size
	Currently Own Pets *n* = 149			No Pets, Owned Pets Before *n* = 31			No Pets, Plan to Own Pets *n* = 233							
	*M*	*SD*	*Med*	*M*	*SD*	*Med*	*M*	*SD*	*Med*	*M*	*SD*	*Med*	*H*, *p* (*df* = 2)	*η* ^2^
Financial burden (e.g., cost of veterinary care, food, supplies, or extra pet fee)	3.67	1.27	4.00 ^a^	4.29	0.86	5.00 ^b^	4.22	0.90	4.00 ^b^	4.02	1.08	4.00	19.21, <0.001	0.042
Housing restrictions (e.g., non-pet-friendly accommodations, less likely to find roommates)	3.54	1.23	4.00 ^a^	4.00	0.93	4.00 ^ab^	4.20	1.01	4.00 ^b^	3.94	1.13	4.00	32.07, <0.001	0.074
Sellers or shelters less willing to sell/adopt because of status as an international student	2.40	1.28	2.00 ^a^	2.90	1.32	3.00 ^ab^	3.16	1.07	3.00 ^b^	2.87	1.22	3.00	34.76, <0.001	0.083
Friends or parents do not support decision to get a pet	2.44	1.34	2.00 ^a^	2.45	1.23	2.00 ^ab^	2.86	1.39	3.00 ^b^	2.67	1.37	3.00	9.12, 0.006	0.018
Concerns about pet care during travel or vacations, such as returning to hometown during college break	4.16	1.01	4.00	4.23	1.18	5.00	4.31	0.93	5.00	4.25	0.98	5.00	2.84, 0.198	0.002
Not sure about future plans, so cannot commit to long-term ownership	2.66	1.45	2.00 ^a^	3.97	1.22	4.00 ^b^	3.83	1.25	4.00 ^b^	3.42	1.44	4.00	60.53, <0.001	0.145
Health concerns for self or others in residence	2.06	1.18	2.00 ^a^	2.38	1.43	2.00 ^ab^	2.51	1.20	2.00 ^b^	2.34	1.23	2.00	13.10, <0.001	0.028

Note: *M*: mean; *SD*: standard deviation; *Med*: median. Scores ranged from 1 (strongly disagree) to 5 (strongly agree). Medians with different superscript letters indicate significantly different mean ranks at *p* < 0.05. Interpreting *η*^2^: 0.01 small, 0.06 medium, and 0.14 large.

**Table 3:** Descriptive Statistics for Ratings of Obstacles in Maintaining Pets in the U.S. by Pet Ownership Status.

	Pet Ownership Status									Total *n* = 413			Kruskal–Wallis Test Results	Effect Size
	Currently Own Pets *n* = 149			No Pets, Owned Pets Before *n* = 31			No Pets, Plan to Own Pets *n* = 233							
	*M*	*SD*	*Med*	*M*	*SD*	*Med*	*M*	*SD*	*Med*	*M*	*SD*	*Med*	*H*, *p* (*df* = 2)	*η* ^2^
Financial burden (e.g., veterinary care, food, supplies, extra pet rent fee)	3.28	1.41	4.00 ^a^	4.10	1.19	5.00 ^b^	4.05	1.09	4.00 ^b^	3.77	1.28	4.00	31.60, <0.001	0.075
Relocating domestically in the U.S. with pets	2.89	1.45	3.00 ^a^	3.43	1.38	4.00 ^ab^	3.62	1.18	4.00 ^b^	3.33	1.35	4.00	23.09, <0.001	0.053
International travel with pets to bring them home	3.41	1.45	4.00 ^a^	4.00	1.08	4.00 ^ab^	4.18	0.98	4.00 ^b^	3.88	1.24	4.00	26.19, <0.001	0.061
Finding reliable and cost-effective pet sitters	3.44	1.43	4.00 ^a^	3.48	1.41	4.00 ^ab^	4.05	1.01	4.00 ^b^	3.78	1.25	4.00	15.80, <0.001	0.035
Uncertainty about future plans prevents commitment to long-term pet ownership	2.62	1.42	2.00 ^a^	4.00	1.23	4.00 ^b^	3.88	1.14	4.00 ^b^	3.42	1.40	4.00	71.73, <0.001	0.177
Pet(s) makes it less likely to build or maintain interpersonal relationships	1.78	1.09	1.00 ^a^	2.28	1.36	2.00 ^ab^	2.25	1.22	2.00 ^b^	2.07	1.20	2.00	16.21, <0.001	0.036
Allergies or other health issues have developed	2.07	1.34	1.00 ^a^	2.10	1.40	1.00 ^ab^	2.39	1.29	2.00 ^b^	2.25	1.33	2.00	8.14, 0.017	0.016
Pets’ behavioral issues	2.27	1.32	2.00 ^a^	2.61	1.50	2.00 ^ab^	2.67	1.19	3.00 ^b^	2.52	1.28	2.00	10.86, 0.004	0.022
Incompatibility between pet(s) and significant other	1.70	1.07	1.00 ^a^	2.03	1.25	2.00 ^ab^	2.43	1.27	2.00 ^b^	2.13	1.24	2.00	35.03, <0.001	0.085
Lost interest in keeping pet(s)	1.39	0.84	1.00 ^a^	1.72	1.03	1.00 ^ab^	1.86	1.14	1.00 ^b^	1.68	1.05	1.00	22.37, <0.001	0.053
Hard to balance pet care and other responsibilities (e.g., studying, etc.)	2.26	1.33	2.00 ^a^	3.07	1.46	3.00 ^b^	3.26	1.27	3.00 ^b^	2.88	1.39	3.00	46.46, <0.001	0.110

Note: *M*: mean; *SD*: standard deviation; *Med*: median. Scores ranged from 1 (strongly disagree) to 5 (strongly agree). Medians with different superscript letters indicate significantly different mean ranks at *p* < 0.05. Interpreting *η*^2^: 0.01 small, 0.06 medium, and 0.14 large.

**Table 4:** Future Plan for Pets.

	Currently Own Pets	No Pets, Plan to Own Pets	Total	Group Differences		Effect Size
	*n* = 148	*n* = 232	*n* = 380			
	*n* (*%*)	*n* (*%*)	*n* (*%*)	*χ*^2^ (*df*)	*p*	Cramer’s *V*
Intended to bring all of their pets with them	112 (75.7%) ^a^	120 (51.7%) ^b^	232 (61.1%)	25.57 (1)	<0.001	0.259
Intended to leave some or all of their pets in the U.S.	8 (5.4%) ^a^	11 (9.2%) ^a^	19 (5.0%)			
Uncertain about their future plans regarding their pets	28 (18.9%) ^a^	101 (43.5%) ^b^	129 (33.9%)			

Note: *n*: frequencies; *%*: percentage. *n* with different superscript letters significantly different at *p* < 0.05. Interpreting Cramer’s *V*: 0.10 small, 0.30 medium, and 0.50 large.

**Table 5:** Descriptive Statistics for Ratings of Attitudes and Perceived Benefits of Pet Ownership by Pet Ownership Status.

					Kruskal–Wallis Test Results		Effect Size
				
	*n*	*M*	*SD*	*Med*	*H* (*df*)	*p*	*η* ^2^
International students should avoid having any pets during their study period in the U.S.	655	2.50	1.14	2.00	39.99 (3)	<0.001	0.057
Currently Own Pets	147	2.13	1.05	2.00 ^a^			
No pets, Owned Pets Before	31	2.52	2.03	2.00 ^ab^			
No Pets, Plan to Own Pets	230	2.39	1.17	2.00 ^a^			
No Plan to Own Pets	247	2.83	1.10	3.00 ^b^			
The benefits of having a pet outweigh the challenges.	407	3.84	0.97	4.00	13.02 (2)	0.001	0.027
Currently Own Pets	147	4.00	1.05	4.00 ^a^			
No pets, Owned Pets Before	30	3.47	1.04	3.00 ^b^			
No Pets, Plan to Own Pets	230	3.78	0.89	4.00 ^b^			

Note: *n*: frequencies; *M*: mean; *SD*: standard deviation; *Med*: median. Scores ranged from 1 (strongly disagree) to 5 (strongly agree). Medians with different superscript letters indicate significantly different mean ranks at *p* < 0.05. Interpreting *η*^2^: 0.01 small, 0.06 medium, and 0.14 large.

## Data Availability

The original contributions presented in this study are included in the article. Further inquiries can be directed to the corresponding author.
